# Transcriptome-based screening of intracellular pathways and angiogenesis related genes at different stages of thiram induced tibial lesions in broiler chickens

**DOI:** 10.1186/s12864-020-6456-9

**Published:** 2020-01-15

**Authors:** Ali Raza Jahejo, Ding Zhang, Sheng Niu, Raza Ali Mangi, Afrasyab Khan, Muhammad Farhan Qadir, Ajab Khan, Huan-chun Chen, Wen-xia Tian

**Affiliations:** 1 College of Animal Science and Veterinary Medicine, Shanxi Agricultural University, Taigu, 030801 China; 2 The State Key Laboratory of Agricultural Microbiology, College of Veterinary Medicine, Huazhong Agricultural University, Wuhan, 430070 China

**Keywords:** Angiogenesis, Broiler, Chondrocytes, Erythrocytes, Thiram

## Abstract

**Background:**

The Tibial dyschondroplasia (TD) in fast-growing chickens is mainly caused by improper blood circulation. The exact mechanism underlying angiogenesis and vascularization in tibial growth plate of broiler chickens remains unclear. Therefore, this research attempts to study genes involved in the regulation of angiogenesis in chicken red blood cells. Twenty-four broiler chickens were allotted into a control and thiram (Tetramethyl thiuram disulfide) group. Blood samples were collected on day 2, 6 (8- and 14-days old chickens) and 15 (23 days old chickens).

**Results:**

Histopathology and hematoxylin and eosin (H&E) results showed that angiogenesis decreased on the 6th day of the experiment but started to recover on the 15th day of the experiment. Immunohistochemistry (IHC) results confirmed the expressions of integrin alpha-v precursor (ITGAV) and clusterin precursor (CLU). Transcriptome sequencing analysis evaluated 293 differentially expressed genes (DEGs), of which 103 up-regulated genes and 190 down-regulated genes were enriched in the pathways of neuroactive ligand receptor interaction, mitogen-activated protein kinase (MAPK), ribosome, regulation of actin cytoskeleton, focal adhesion, natural killer cell mediated cytotoxicity and the notch signalling pathways. DEGs (*n* = 20) related to angiogenesis of chicken erythrocytes in the enriched pathways were thromboxane A2 receptor (*TBX*A2*R*)*,* interleukin-1 receptor type 1 precursor (*IL*1*R*1)*,* ribosomal protein L17 (*RP*L17)*,* integrin beta-3 precursor (*ITG*B3)*, ITG*AV*,* integrin beta-2 precursor (*ITG*B2)*,* ras-related C3 botulinum toxin substrate 2 (*RAC*2)*,* integrin alpha-2 (*ITG*A2)*,* IQ motif containing GTPase activating protein 2 (*IQGAP*2)*,* ARF GTPase-activating protein (*GIT*1)*,* proto-oncogene vav (*VAV*1)*,* integrin alpha-IIb-like (*ITG*A5)*,* ras-related protein Rap-1b precursor (*RAP*1B)*,* tyrosine protein kinase Fyn-like (*FYN*)*,* tyrosine-protein phosphatase non-receptor type 11 (*PTPN*11)*,* protein patched homolog 1 (*PTCH*1)*,* nuclear receptor corepressor 2 (*NCOR*2) and mastermind like protein 3 (*MAML*3) selected for further confirmation with qPCR. However, commonly DEGs were sarcoplasmic/endoplasmic reticulum calcium ATPase 3 (*ATP*2A3), ubiquitin-conjugating enzyme E2 R2 (*UBE*2*R*2), centriole cilia and spindle-associated protein (*CCSAP*), coagulation factor XIII A chain protein (*F*13A1), shroom 2 isoform X6 (*SHROOM*2), ras GTPase-activating protein 3 (*RAS*A3) and *CLU*.

**Conclusion:**

We have found potential therapeutic genes concerned to erythrocytes and blood regulation, which regulated the angiogenesis in thiram induced TD chickens. This study also revealed the potential functions of erythrocytes.

**Graphical abstract:**

1. Tibial dyschondroplasia (TD) in chickens were more on day 6, which started recovering on day 15. 2. The enriched pathway observed in TD chickens on day 6 was ribosome pathway, on day 15 were regulation of actin cytoskeleton and focal adhesion pathway. 3. The genes involved in the ribosome pathways was ribosomal protein L17 (*RP*L17). regulation of actin cytoskeleton pathway were Ras-related C3 botulinum toxin substrate 2 (*RAC2*), Ras-related protein Rap-1b precursor (*RAP*1B), ARF GTPase-activating protein (*GIT*1), IQ motif containing GTPase activating protein 2 (*IQGAP*2), Integrin alpha-v precursor (*ITG*AV), Integrin alpha-2 (*ITG*A2), Integrin beta-2 precursor (*ITG*B2), Integrin beta-3 precursor (*ITG*B3), Integrin alpha-IIb-like (*ITG*A5). Focal adhesion Proto-oncogene vav (Vav-like), Tyrosine-protein kinase Fyn-like (*FYN*). 
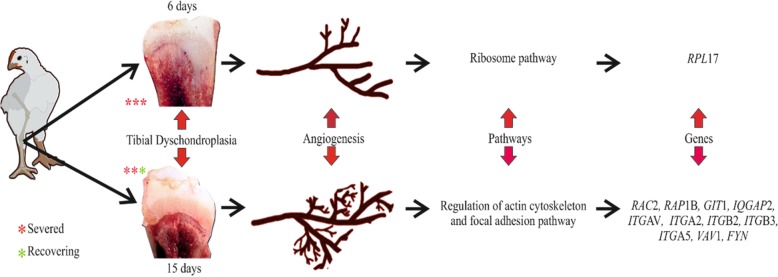

## Background

Tibial dyschondroplasia (TD) is a bone deformity disease characterized by accumulation of non-mineral and avascular cartilage that causes chondrocyte death due to lack of blood supply [1, 2]. The death of blood vessels membrane is called angionecrosis, which reduces angiogenesis in broilers and causes TD [3]. It has been reported that high level of blood vessels formation increase the size of the growth plate and reduce the chance of TD [4]. Thiram is a pesticide that is commonly used in agriculture. It may cause lameness and cartilage defect in avian [2, 5]. Previous studies have demonstrated that thiram induces proliferation and disturbs the endochondral calcification in the growth plate of broiler chickens [6]. However, it is also reported that red blood cells have a direct role in the immune response and have an indirect effect on certain pathogens and pro-inflammatory factors [7, 8]. A study also showed that tibial angiogenesis in the hypertrophic zone was strongly suppressed by thiram-induced TD [9]. These clues suggest that the development of TD may be related to the immune response of red blood cells in chicken blood vessels. Previously, we reported that the transcripts of eight avian β-defensins (AvBDs) and liver-expressed antimicrobial peptide 2 (LEAP-2) found in chicken erythrocytes participated in Mareks disease virus (MDV) induced host immune response [10]. Erythrocytes also play a vital role in the immune response to TD chickens by upregulating the host defense peptides (HDPs) [11]. These findings raise the possibility that immune responses of chicken erythrocytes contribute to prevent and treat TD. Whereas, the angionecrosis has been proved to be the cause of thiram induced TD and most of the literature confirms the detrimental effects of angiogenesis [2–4]. However, the mechanisms underlying the angionecrosis leading to TD has not been well studied. Therefore, the current study of transcriptome sequencing investigated the potential target genes of angiogenesis in chicken erythrocytes of thiram induced TD. These genes regulate blood circulation and angiogenesis, providing a new insight into the therapeutic manipulation in order to prevent TD.

## Results

### Histopathology

**Fig. 1:**
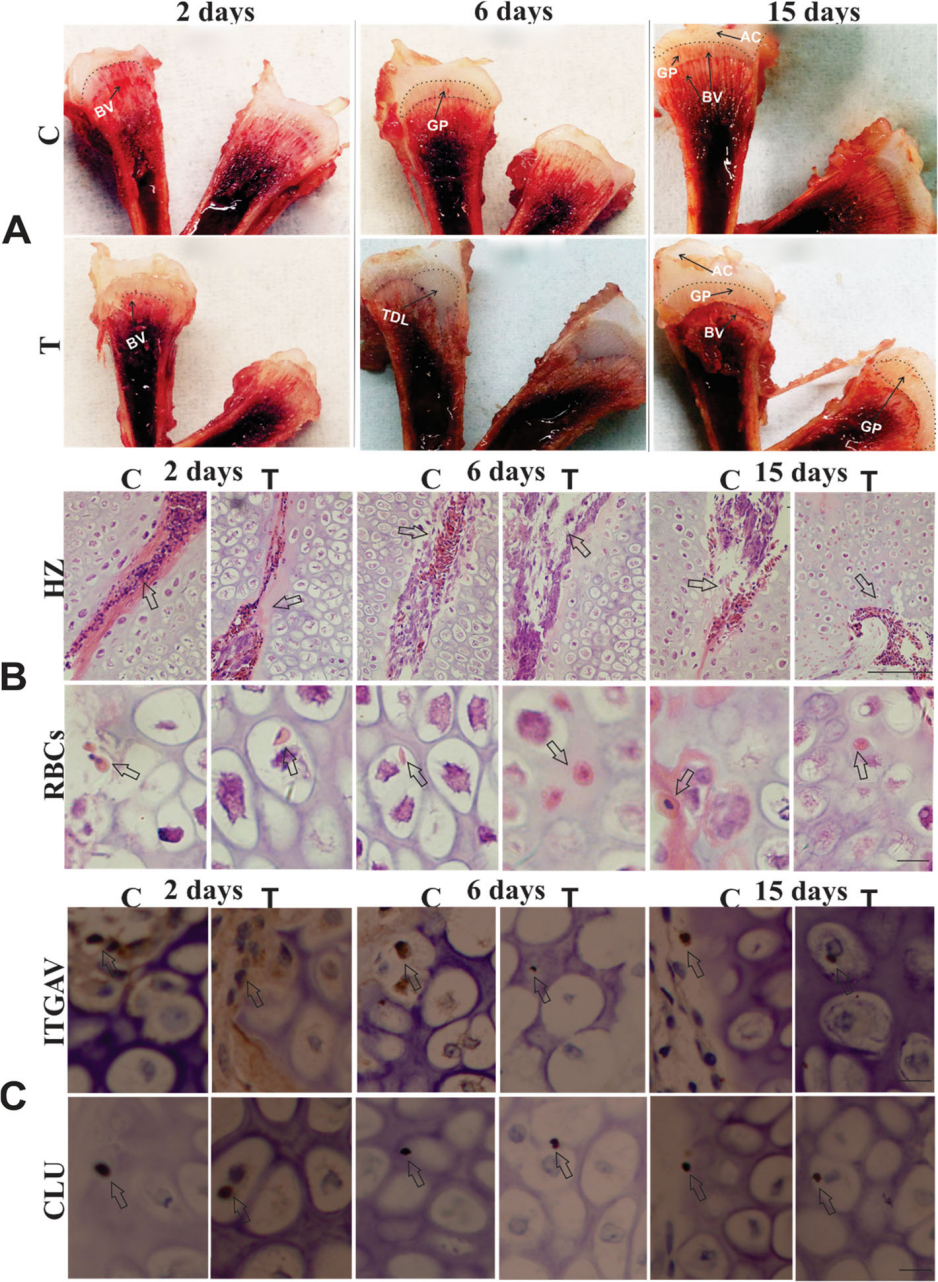
Morphology, histology and immunohistochemistry (*n* = 4). **a** The morphological observation of tibia, on lesion development and blood vessel distribution in the tibiotarsus of chicken. AC = articular cartilage; GP = growth plate; BV = blood vessels; TDL = tibial dyschondroplasia lesions. **b** H&E stained histopathology of the tibial growth plate (GP). The chondrocytes were organized, and growth plate shows regular columns of cells surrounded by blood vessels in the hypertrophic zone of the control group and reduced number of blood vessels in the thiram group (Day 2, 6 and 15). The chondrocytes were damaged, and necrosis was observed. Most importantly erythrocytes were seen in the chondrocytes of control and thiram-fed chickens. Arrows in HZ indicate the blood vessels. HZ = hypertrophic zone (Bar-100 μm). Arrows in RBCs shows the red blood cells which were seen within/around chondrocytes. RBCs = red blood cells (Bar-50 μm), C = control, T = thiram. **c** Immunohistochemical localization of ITGAV and CLU in control and thiram groups. The control group on day 15 has more localization of ITGAV than thiram group. The thiram group on day 15 has higher localization of CLU which decreased on day 15 in the control group. Arrows indicate the expression of ITGAV and CLU in red blood cells which were seen within/around chondrocytes. T = thiram, C = control. (Bar-50 μm)

The chickens (*n* = 4) in a control group were active, and healthy. However, the chickens (n = 4) in thiram group had lesions in tibiotarsus bone. The lameness began on day 2, became more visible on day 6, and decreased on day 15. More lesions and few blood vessels were seen in the growth plate of thiram-fed chickens on day 2. The lesions were severed on day 6 and relatively decreased on day 15 (Fig. 1a). The differences within the groups turned out to be more prominent and obvious, because the blood vessels began to recover in the thiram group on day 15 as compared to day 6, when some area of the growth plate had been calcified and vascularized after the severity of lesions.

The histological examination of the hypertrophic zone of ​​the growth plate was observed under a microscope. The chondrocytes were in a columnar arrangement, and the blood vessels in the control group were normal. The proliferation of chondrocytes was affected, as their size and shape were degraded. These arrangements were less disordered on day 2, more on day 6, and began to recover on day 15 in thiram group. Most importantly, the erythrocytes could be clearly seen in and around the chondrocytes of the control and thiram groups (Fig. 1b).

Denser erythrocytes were found on day 2, lighter on day 6, and prominent recovery was seen on day 15 (Additional file 4: Figure S1). However, the quantification of microscopic figures with ImageJ software suggests that the area and the density of blood vessels started improving on day 15, (Additional file 4: Figure S2 A and B).

Expression of CLU and ITGAV in chicken red blood cells was examined by IHC analysis (Fig. 1c). The quantification results by ImageJ software with IHC profile suggest that as compared to the control group, expression of CLU was increased in the thiram group on day 2 (Additional file 4: Figure S3 A, S2 C). Whereas the expression of ITGAV appeared less in number in thiram group as compared to the control group on day 15 (Additional file 4: Figure S3 B, S2 C).

### Differentially expressed genes

**Fig. 2:**
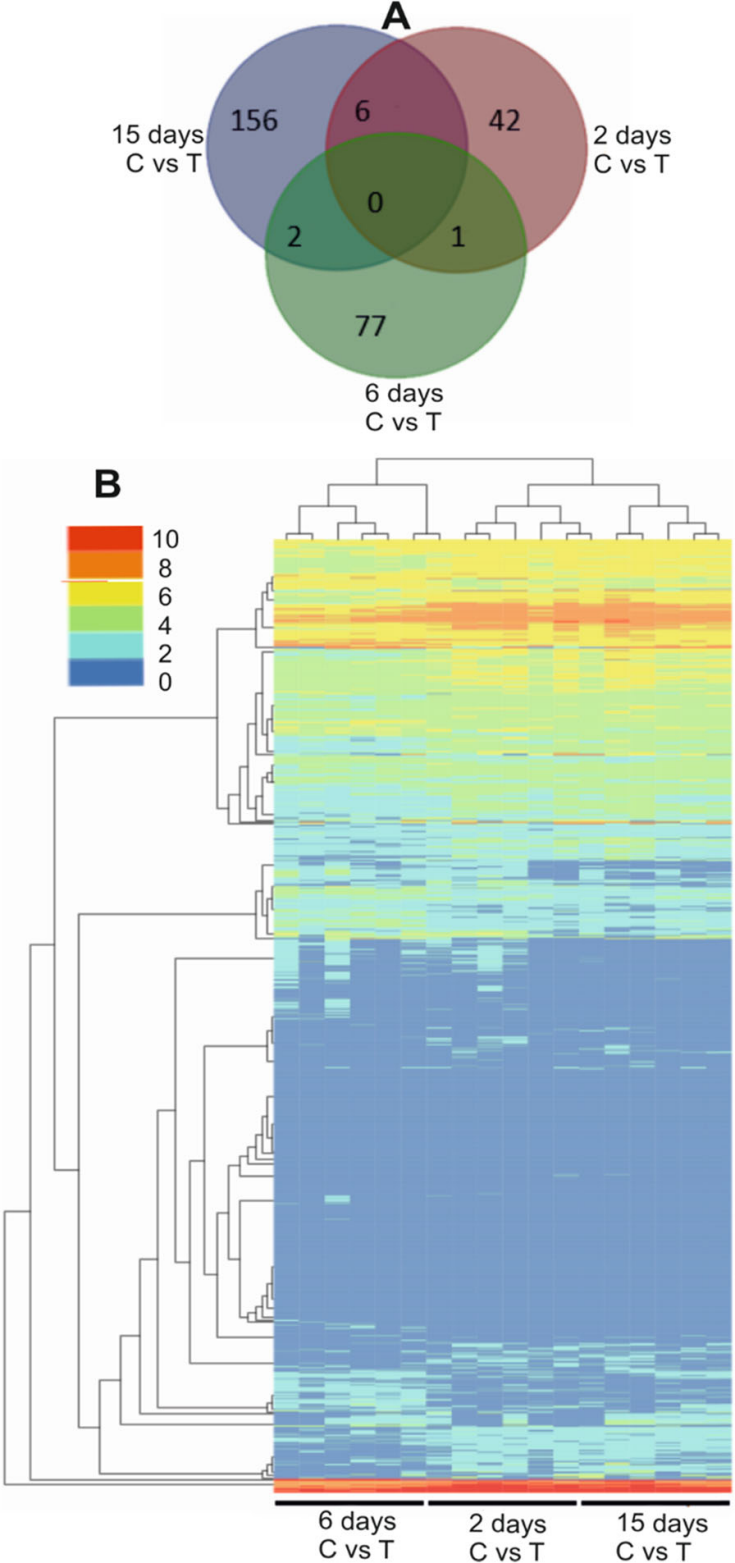
Differential expression of genes (*n* = 3). **a** Analysis of DEGs, using Venn diagrams. There were 49, 80 and 164 DEGs identified on day 2, 6 and 15 respectively. Only a single gene expressed commonly, on day 2 and 6. Total 6 genes were expressed commonly on day 2 and 15. Whereas the total number of genes expressed commonly on day 6 and 15 were 2. **b** Hierarchical clustering analyses of 49, 80 and 164 transcripts on day 2, 6 and 15. The numbers 0, 2, 4, 8 and 10 represent high to low expressions. The vertical axis represents one chicken and horizontal axis refers to a gene

The analysis of differentially expressed genes (*n* = 3) on day 2, 6 and 15 revealed that 293 genes were differentially expressed (*P* < 0.05). The number of differentially expressed genes on day 2, 6 and 15 were 49 (39^↑^up, 10^↓^down), 80 (49^↑^up, 31^↓^down), and 164 (15^↑^up, 149^↓^down), respectively (Fig. 2a. Additional file 1). Hierarchical cluster analysis also showed that chickens on day 6, 2 and 15 formed more gene expression patterns, respectively. The gene expression patterns could be seen similar on day 2 and 6 (Fig. 2b).

We selected some potential genes which were expressed commonly in the groups. The commonly observed gene *UBE*2*R*2 was downregulated on day 2 and upregulated on day 6. Other genes such as, *ATP*2*A*3, *F*13*A*1, *SHROOM*2, *RASA*3 and *CLU* were upregulated on day 2 and downregulated on day 15. The only gene upregulated on both 2 and 15 days was *CCSAP* (Additional file 2: Table S1).

### GO annotations

**Fig. 3:**
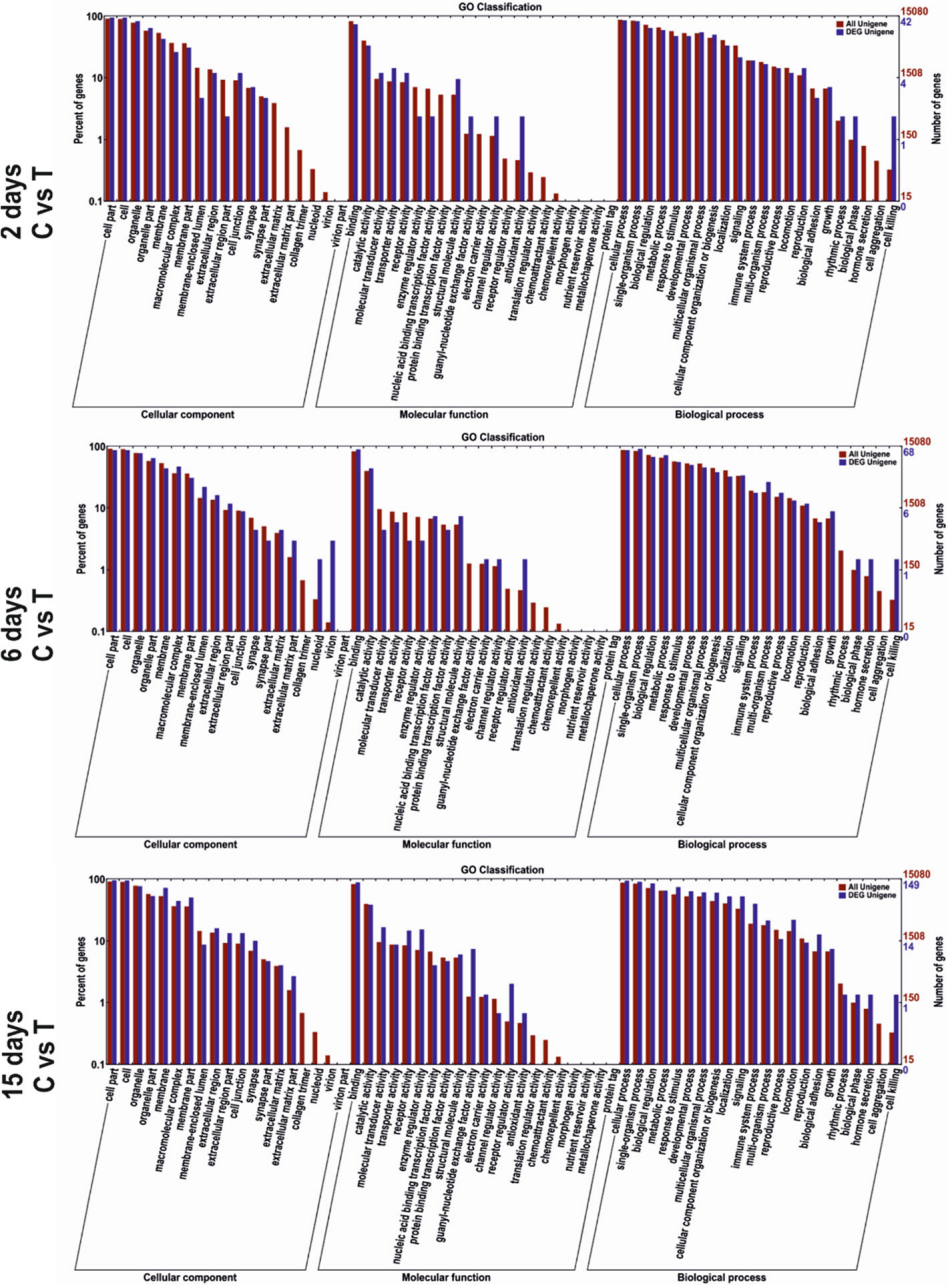
Gene ontology classifications. The differentially expressed genes were assigned in three categories of GO classifications: Cellular component, molecular function, and biological process. C = control, T = thiram

On days 2, 6 and 15, a total of 49, 80 and 164 DEGs were respectively assigned to the gene ontology (GO). GO enrichment analysis was performed from three aspects of cellular component, molecular function and biological process. Among these categories, most DEGs were enriched in the cellular component category. Within the cellular component category, cell and cell part were the most dominant subcategories. Regarding the molecular function category, the four most abundant sub-categories were binding, catalytic activity, molecular transducer activity, and transporter activity. As for the biological process category, the most DEGs were assigned to a cellular process, single organism process, biological regulation, and metabolic process (Fig. 3).

### Analysis of COG enrichment

**Fig. 4:**
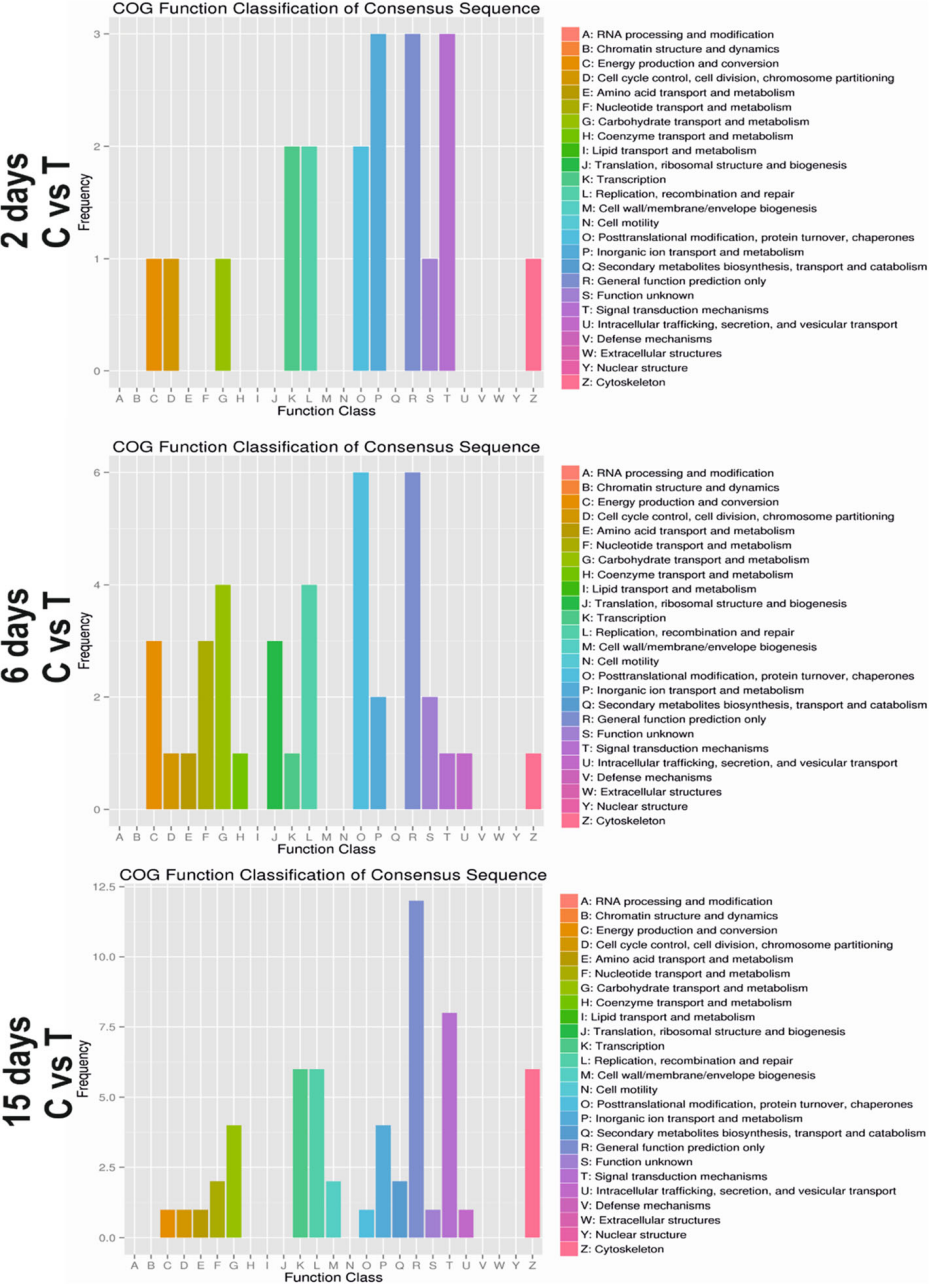
COG function classification. The COG function classification of the consensus sequences. The number of genes shown on horizontal and proportions plotted on the vertical axis. C = control, T = thiram

Annotations of differentially expressed genes against the COG data base showed 3, 6 and 12 DEGs which could not be annotated accurately on day 2, 6 and 15 respectively and were classified in the category of general function prediction only. Based on the number of annotations genes, the second top category on 2 and 15 days was the signal transduction mechanism. However, on day 6 the second top category was Posttranslational modification, protein turnover, chaperones according to the COG database, which suggests that DEGs in the groups of these days may play important roles in TD (Fig. 4).

### Analysis of potential KEGG pathways

**Fig. 5:**
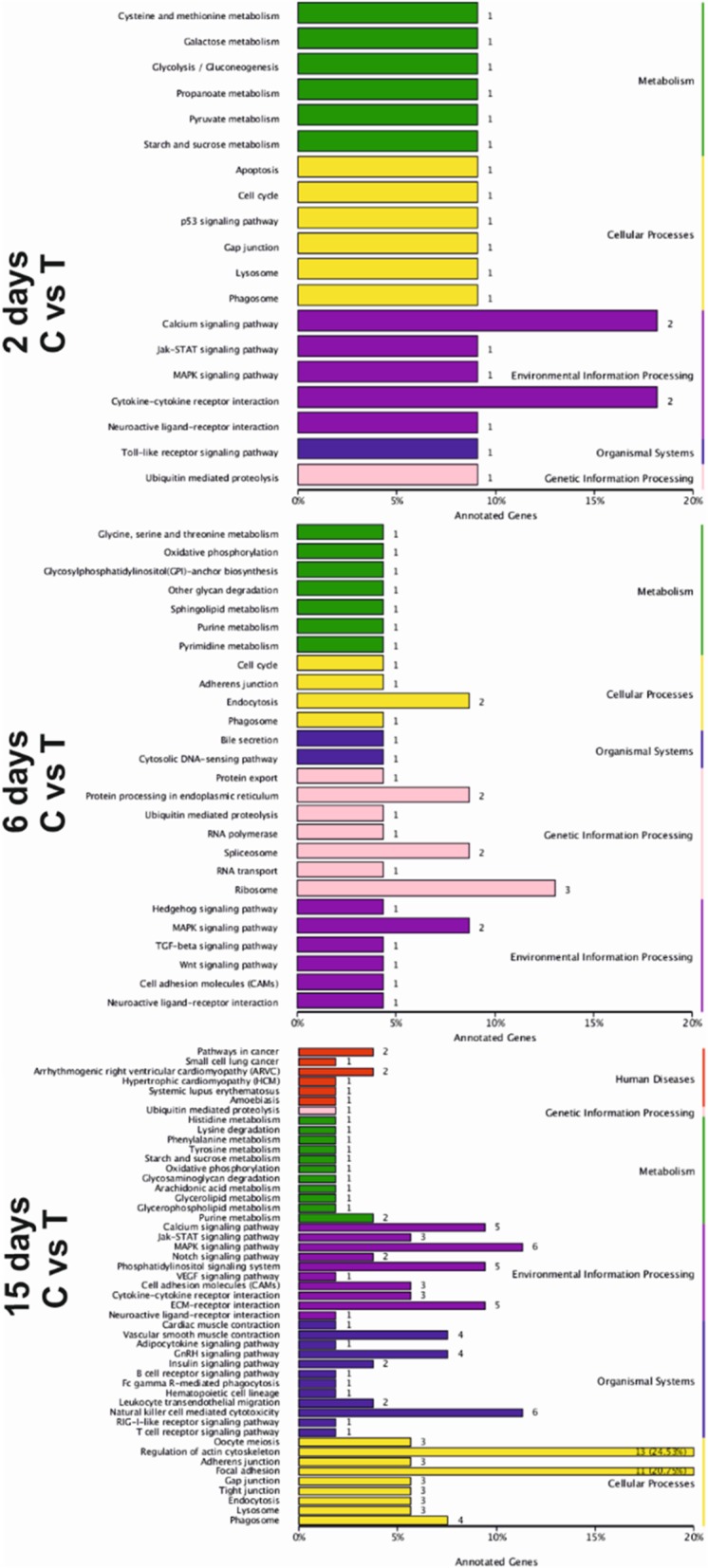
KEGG categories. The KEGG categories of DEGs. The vertical axis shows the metabolic pathways and the horizontal axis shows the proportion of annotated genes. C = control, T = thiram

We analysed the regulatory pathways of the DEGs using KEGG analysis. The potential KEGG pathways of DEGs on day 2, 6- and 15 are shown in Fig. 4. The KEGG pathways in which most DEGs were enriched on day 2 were calcium signaling pathway, cytokine receptor interaction, MAPK signaling pathway and neuroactive ligand receptor interaction pathway. The KEGG pathways in which most DEGs were enriched on day 6 were the ribosome pathway and MAPK signaling pathway. Most DEGs enriched on day 15 in KEGG pathways were regulation of actin cytoskeleton pathway and focal adhesion pathway (Fig. 5, Additional file 4: Figure S4). Moreover, the angiogenesis related pathways found on day 2, 6 and 15 were hedgehog signaling pathway, vascular endothelial growth factor signaling pathway (VEGF) and the Notch signaling pathway (Additional file 4: Figure S4). We selected the top 20 angiogenesis related genes belonging to these pathways for further confirmation under qPCR experiment.

### Screening of DEGs and qPCR

Screening of differentially expressed angiogenesis genes were done based on their enrichment in the KEGG pathways, COG and GO annotations. Selected DEGs for further confirmation under qPCR experiment were *CLU, F*13*A*1, *TBXA*2*R*, *IL1*R1, *RP*L17, *RAC*2, *RAP*1B, *GIT*1, *IQGAP*2, *ITG*AV, ITG*A*2, ITG*B*2, *ITG*B3, *ITG*A5, Vav-like, *FYN*, *PTPN*11, *NCOR*2, *MAML*3 and *PTCH*1 (Additional file 3: Table S2).

**Fig. 6:**
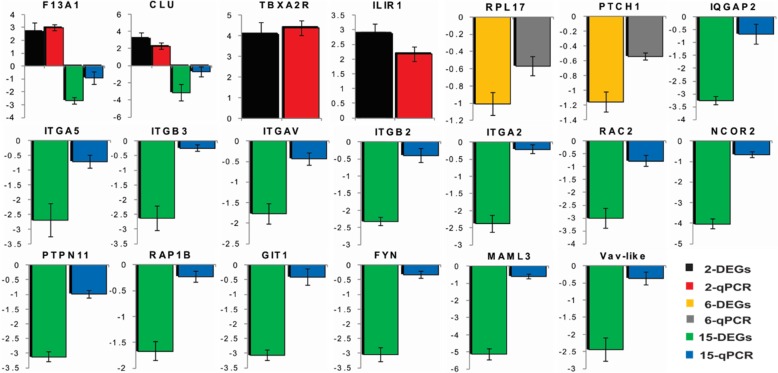
Validation of DEGs. The validation of DEGs by qPCR (n = 3, same as RNA-seq). All the gene expressions were validated with DEG’s data on 2, 6 and 15 days. 2-DEGs = differentially expressed genes for 2 days, 2-qPCR = qPCR for 2 days, 6-DEGs = differentially expressed genes for 6 days, 6-qPCR = qPCR for 6 days, 15-DEGs = differentially expressed genes for 15 days, 15-qPCR = qPCR for 6 days

The angiogenesis related DEGs (*n* = 20) were analysed by qPCR for further confirmation (*n* = 3, same as RNA-seq). The results revealed that all 20 genes were validated with DEGs data (Fig. 6). The level of expressions of *F*13*A1, CLU, TBXA*2*R* and *IL1R*1 genes were upregulated in the thiram group on day 2. The DEGs *RP*L17 and *PTCH*1 were downregulated in the thiram group on day 6. Whereas on day 15, *ITG*B3*, ITG*AV*, ITG*B2*, RAC2, ITG*A2*, IQGAP*2*, GIT*1*, VAV*1*, ITG*A5*, RAP*1B*, FYN, PTPN*11*, NCOR*2*,* and *MAML*3 genes were downregulated in the thiram group. Transcriptome sequencing and qRT-PCR results of 20 differentially expressed angiogenesis related genes observed in the same range for day 2 and 6. In addition, the transcriptome sequencing result of day 15 is also consistent with qRT-PCR result but the range of fold change does not follow each other.

## Discussion

In this study, the differentially expressed angiogenesis-related genes of erythrocytes were identified and investigated on different stages of thiram induced TD in broilers. DEGs were screened by transcriptome sequencing on day 2, 6 and 15. Subsequently, 293 differentially expressed genes appeared on day 2 (49 DEGs), day 6 (80 DEGs) and day 15 (164 DEGs), respectively. Secondly, the functions of DEGs were annotated by COG, GO, and KEGG databases and 20 angiogenesis related DEGs and their enrichment pathways were analysed to elucidate the role of angiogenesis and erythrocytes in thiram induced TD. Finally, the RNA sequence was validated by qPCR.

Previously, transcriptome sequencing in TD was mainly focused in early stage [2], but this study reported early and late changes of erythrocytes in thiram induced TD. Thiram is lipophilic in nature and combined with the cell membrane to cause cytotoxicity, bone formation disorder, cartilage cell damage and immune depressions. It can also cause membrane damage, bone morphogenic inactivation and inhibit angiogenesis [12, 13]. In TD, a large number of the cartilage cell damage is due to the apoptosis process and abnormal protein secretion in the cartilage cells, causing a decrease in the cartilage extracellular matrix degradation rate, which limits the space for bone deposition [14, 15]. The cells stop to multiply and endure hypertrophy and apoptosis in tibia bone. Thiram destroyed the regular column of chondrocytes and the number of cells is decreased in the tibial growth plate of chickens [13]. In TD, the cells in hypertrophic zone were degraded, dead and arranged in irregular columns, having no nucleus in it [16]. This study showed that there were more lesions and fewer blood vessels in the growth plate of thiram-fed chickens. The blood vessels started sprouting in the thiram group on late stages when some area of the growth plate had become calcified and vascularized after the severity of lesions. Hematoxylin and eosin result also revealed that the proliferation of chondrocytes was affected, as their size and shape were degraded, and arrangements were disordered in the early stage, and recovered at a later stage. The blood vessels were severely affected at an early stage and started getting recover later. This research found the erythrocytes in the chondrocytes of broiler chickens that is a rare case of its type, so we designed this study to guess the function of erythrocytes concerned to angiogenesis-related genes of erythrocytes.

The differentially expressed common genes on day 2, 6 and 15 were *UBE*2*R*2*, CCSAP, ATP*2A3*, F*13*A*1*, SHROOM*2*, RASA*3*,* and *CLU*. Firstly, *UBE*2*R*2 noticed significantly downregulated on day 2 and upregulated on day 6. Furthermore, COG categories of function classification regarding protein turnover was enriched on day 6, which guess that thiram induce TD increases the level of *UBE*2*R*2 to degrade the proteins in the chickens. Previous research supports our findings, as ubiquitin conjugating enzymes including *UBE*2*R*2 is responsible to degrade the proteins through proteasome [17]. HIF-1α is one of the major client proteins of heat-shock protein 90 (Hsp90), and it is required for the functioning and the rapid hypoxic stabilization of HIF-1 α, which is degraded by the ubiquitin-proteasome protein system [18]. In addition, this is the first finding related to *UBE*2*R*2 in thiram induced TD chickens. Secondly, *ATP*2*A*3 was upregulated on day 2 and downregulated on day 15, which have been evaluated previously as a transporter of Ca^+ 2^ ions [19]. A recent study reported that Ca^+ 2^ ions decreased in TD chickens [20]. KEGG pathways results of our study demonstrated that calcium signaling pathway was not enriched and differentially expressed on day 6, which was the clear indication of decreased or less Ca + ions transportation compared on day 2 and 15. The results of this study supported and added in the findings of Huang et al.*,* [20], thiram significantly downregulated the expression of *ATP2A3*, which could be responsible for the reduction of Ca^+ 2^ ions transportation similarly in TD chickens. Moreover, calcium signaling pathway shows that the chickens are being recovered from the deleterious effect of thiram on day 15. Thirdly, *Shroom2* was upregulated on day 2 and downregulated on day 15. It has an important role to develop vasculature, which is required for proper angiogenesis; reduction in which decrease the endothelial contractility and cause sprouting [21]. Fourthly, *RASA3* is a GTPase activating protein of the GAP1 family, targets the R-Ras and Rap1 [22]. Rap1 signaling is important for angiogenesis from pre-existing ones [23]. *RASA*3 have been found upregulated on day 2 and downregulated on day 15, although not differentially expressed on day 6. It can be illustrated that angiogenesis is developing on day 2, in contrast to day 6 and sprouting developed from pre-existing ones on day 15. Fifthly, *CLU* defends the apoptosis and necrosis of cells induced by the genotoxic and oxidative stress [24]. In our study, *CLU* upregulated on day 2 and downregulated on day 15. Whereas on day 6, it was not differentially expressed. Sixthly, F13A1 is a key molecule, which coagulates the blood and, played role in angiogenesis and tissue repairing when interacts with vascular endothelial growth factor receptor 2 (VEGFR2) and integrin αVβ3 for optimal tissue healing [25]. Seventhly, *CCSAP* is the only gene which differentially expressed and upregulated on both 2 and 15 days. Its role is not cleared yet, even though very limited literature is available which demonstrated that it localizes to polyglutamylated microtubules and promotes proper cilia function and embryonic development [26]. Another study reported that proper expression of CCSAP is required for normal mitotic progression [27]. Based on the results of our study and previous reports, it could be demonstrated that *CCSAP* is responsible for the stability of erythrocytes in thiram induced TD chickens.

The annotation function of differentially expressed genes in the KEGG database explored 7 enriched pathways with most DEGs on 2, 6 and 15 days were neuroactive ligand receptor interaction, MAPK signaling, ribosomes, regulation of actin cytoskeleton, focal adhesion and natural killer cell mediated cytotoxicity and Notch signaling pathways. In this study, we elaborated and predicted the role of above-mentioned pathways in angiogenesis of thiram fed chickens; including some notable DEGs to identify the potential pathways and candidate genes.

The neuroactive ligand-receptor interaction and MAPK signaling pathway were found significantly enriched on day 2, including DEGs *TBXA*2*R* and *IL1R*1. Recent research reported *TBXA*2*R* a novel cancer gene, involved in aggregation and activation of platelets, which activate multitude of signaling cascades to control various cellular processes, like vasoconstriction of smooth muscle, responses to inflammation, cell adhesion, motility, proliferation, and the cell survives; showed dramatic cell killing when decreased/downregulated the level of *TBXA*2*R* [28, 29]. *IL1R*1 is the cell surface receptor, which is mediators of inflammation, controls the reactions produced in response to injury or stress. The upregulation of *IL1R*1 indicated osteoarthritis [30]. This study predicted the expressions of MAPKs started increasing on day 2, in response to stress/injury caused by thiram. It could be stated that the harmful effect of thiram induced TD in chickens started on day 2. This study agreed to a recent transcriptional study of Malgulwar et al*,* [31] who concluded that *IL1R*1 could play a role in phenotypic changes of high microvascular densities and increased expressions of VEGF.

The ribosome pathway has been reported to be related to cell growth, proliferation, and apoptosis [32]. It mediated the angiogenesis in response to injury or stress. *RP*L17 acts as tumor inhibition. If the expression of *RP*L17 downregulated, it causes the proliferation of VSMC [33]*.* Immunohistochemistry and H & E staining results of this study evaluated those blood vessels destructed and diminished in thiram group on day 6 as compared to all other groups. It could be stated that thiram significantly downregulated the expressions of ribosomal pathway gene *RP*L17 on day 6 which resultantly reduced the angiogenesis. The relationship between ribosomal protein functions and angiogenesis has not been studied till date in thiram fed chickens. This is the first ever study of its type, in which the role of *RP*L17 is elaborated as concerned to thiram fed chickens. Concerned to ribosomal proteins, these results could be valuable for further investigation of angiogenesis and erythrocytes in TD. Formerly, it has already been reported that *RP*L17 gene might play a role in suppressing angiogenesis [32]*.* In addition, *RP*L17 found significantly downregulated in chicken, which causes remodeling through vascular smooth cell proliferation and inflammation [34].

The actin cytoskeleton and focal adhesion pathway are crucial for the cell adhesion, cell motility, and morphological changes, which may impact angiogenesis and cell adhesion to protect the cells from various toxic mediators [35, 36]. The actin cytoskeleton plays an essential role in maintaining normal functions of a cell by modulating the shape, migration, and proliferation, which are needed for angiogenesis [35].

Transcriptome sequencing results of this study identified several DEGs, involved in the actin cytoskeleton and cell adhesion pathways on day 15 were *RAC2, RAP*1B*, GIT*1*, IQGAP*2*, ITG*AV*, ITG*A2*, ITG*B2*, ITG*B3*, ITG*A5*, VAV1, FYN.* DEGs, *RAC*2*,* and *RAP*1B belong to ras family, whereas GIT1 and IQGAP2 belong to GTPase-activating protein family. Previously, it has also been reported that Ras family and GTPase activating protein family regulate angiogenesis, with controlling the endothelial cell functions [37].

Integrins are the transmembrane receptors which mediate cell extracellular matrix (ECM) adhesion. On ligand binding, integrins activate the signal transduction pathways which facilitates cellular signals such cell cycle regulation, an organization of the intracellular cytoskeleton, and the movement of new receptors to the cell membrane [38]. In our study, the selected enriched DEGs on day 15 were *ITG*AV*, ITG*A2*, ITG*B2*, ITG*B3*, ITG*A5, which have been defined as integrin family [39], involved in the cytoskeleton and focal adhesion pathway of this study. Integrins took part in the angiogenesis and considered a potential antiangiogenic target which up-regulated in tumor related blood vessels [40, 41]. The results of this study are in line with recent studies of Khalid Mehmood et al.*,* [42], who examined the expression of *ITG*B3 in thiram treated chickens and found downregulation of *ITG*B3 gene on day 14. Similarly, we have found *ITG*B3*,* including *ITG*AV*, ITG*A2*, ITG*B2, and *ITG*A5 integrin genes downregulated in TD-effected chickens on day 15 as compared to normal chickens. Khalid Mehmood *et., al* [42] has found that, the usage of ligustrazine in TD chickens significantly increased the expression of ITGB3 which helps to prevent the TD. We found five integrin family downregulated genes on day 15 of thiram-induced TD chickens-including, *ITG*B3 and other integrins such as *ITG*AV*, ITG*A2*, ITG*B2*,* and *ITG*A5 may have a crucial role in angiogenesis. This is the first study on thiram induced TD chickens, which reported the role of integrin family genes in angiogenesis, future research concerned to integrin genes should be focused on it to develop medication in response to increase expressions in TD chickens.

Natural killer (NK) cell-mediated cytotoxicity pathway contributed to the innate immunity to response numerous malignancies, including leukemia [43]. We have found *VAV*1*, FYN, PTPN*11 genes involved in (NK) cell-mediated cytotoxicity pathway downregulated in thiram induced TD chickens on day 15. The physiological function of Vav-like gene is restricted to the hematopoietic system, where it plays a critical role in the development and activation of T-cells. Fyn tyrosine kinase participates in several biological processes, such as cell growth and differentiation, it also involved in the pathogenesis of hematologic malignancies [44, 45]. *PTPN*11 is one of the few known phosphatases that can function as an oncogene [46] to dephosphorylate the signaling molecules including Fyn, and Vav1, and terminated the activity of signaling receptors in NK cells [47]. The main function of *PTPN*11 is to suppress the tumor [48]. *PTPN*11 could play a vital role in promoting angiogenesis in TD chickens, if the expressions of *PTPN*11 have been upregulated with medication.

The Notch signaling pathway plays an important part in skeletal development and homeostasis. Serious skeletal disorders can be attributed to alterations in the Notch signaling pathway [49]. Notch pathway inhibits the chondrogenesis and arrests chondrocyte differentiation in chicken embryos and prevents the formation of the hypertrophic zone in the growth plate [50]. *NCOR*2 and *MAML*3 genes downregulated in the Notch signaling pathway on day 15 in our study. Notch receptor signaling controls the endothelial cell proliferation, adhesion, migration and formation of new blood vessels as angiogenesis [51]. *MAML*3 is a critical transcriptional co-activator in the Notch signaling pathway, and is encoded by a family of Mastermind like (*MAML*) genes [52]. *MAML*3 encodes transcriptional co-activators for the Notch signaling pathway [52]. *NCOR*2 also known as silencing mediator of retinoid and thyroid hormone (SMRT) is a potent regulator of retinoid and thyroid hormone signalling [53] which repress inflammatory response genes [54]. The role of *NCOR*2 in TD chicken is still unclear, but we predicted that Notch signaling pathway gene *NCOR*2 repress the inflammatory response of thiram in TD chickens.

## Conclusion

In this study, we have found potential target genes concerned to angiogenesis and erythrocytes which control the blood circulation. To prevent TD in broiler chickens, some therapeutic interventions are needed to regulate these genes, such as *ATP*2*A*3*, UBE*2*R*2*, CCSAP, F*13*A*1*, SHROOM2, RASA3,* and *CLU,* which expressed commonly in the erythrocytes within the trial groups. DEGs (*TBXA*2*R, IL1R*1*, RP*L17*, ITG*B3*, ITG*AV*, ITG*B2*, RAC*2*, ITG*A*2, IQGAP*2*, GIT1, VAV1, ITG*A5*, RAP*1B*, FYN, PTPN11, PTCH1, NCOR2, MAML3*) of potential pathways (Neuroactive ligand receptor interaction, MAPKs, ribosomes, regulation of actin cytoskeleton, focal adhesion, natural killer cell mediated cytotoxicity and the Notch signaling pathway) have important role in angiogenesis concerned to thiram induced TD chickens, most importantly integrin family, ribosome pathway and *RP*L17 gene. However, future research is needed to make medicine which can regulate these genes.

## Methods

### Induction of TD and blood collection

One day old broiler chickens (*n* = 24) were purchased from Shanxi Daxiang Farming Group (Shanxi, China), and fed ad-libitum basal diet for 7 days. After overnight fasting on seventh day, all the 24 broilers were randomly allotted into the control and the thiram groups. The broilers in thiram groups were fed a diet containing 100 mg/kg thiram for 48 h to induce TD [2]. Four birds from each group were sacrificed by cervical dislocation under euthanasia on day 2, 6, and 15 after the thiram feed. Day 2 and 6 (8- and 14-days old chicken) were differentiated as early stage TD chickens, and day 15 (23 days old chickens) were differentiated as late stage TD chickens.

The broilers were bled (2.5 mL) from a brachial vein on day 2, 6 and 15 to collect blood samples and then sacrificed [55]. Before exsanguination and necropsy, injection of pentobarbital was used in conjunction with the standard protocols of euthanasia to minimize suffering. All the procedures complied with the welfare guidelines approved by the College of Animal Science and Veterinary Medicine of Shanxi Agricultural University, China (Number 88, 2010).

### Morphology, histology, and immunohistochemistry

The experimental chickens (*n* = 4) were sacrificed on day 2, 6 and 15 after thiram feed. The tibiae from allotted chickens were immediately taken out after the chickens were sacrificed. Then, left sagittal sections of the proximal tibial growth plate were prepared for morphological analysis [56]. Right proximal tibiae were fixed in 4% paraformaldehyde and H&E staining was performed as mentioned in our previous article [2]. Furthermore, IHC was used for analysing the expression of angiogenesis-related genes integrin alpha-v precursor (ITGAV) and clusterin precursor (CLU), which were deparaffinized in xylene, and rehydrated through series of graded ethanol solutions, rinsed in distilled water, and incubated in 3% H_2_O_2_ [57]. ITGAV and CLU were detected and localized at the cellular level by IHC with rabbit anti-integrin and anti-clusterin (BS9178; Bio world, Nanjing, China). The Immunohistochemistry (IHC) scoring system was used to determine the scores of ITGAV and CLU expression [58]. Furthermore, the quantification of H&E staining was analysed in ImageJ software. The relative area of blood vessels determined in percentage by dividing area of interest of blood vessels with total area (Pixels 4080 × 3072), and the density of blood vessels were measured using ImageJ 1.42q software.

### RNA extraction, library construction, and RNA-sequencing

To extract RNA, the chicken blood samples (2.5 mL) of control and thiram groups (*n* = 3 randomly) were collected on day 2, 6 and 15. Total RNA was extracted from the erythrocytes of each group on day 2, 6 and 15 using TRIzol reagent (Invitrogen, Carlsbad, CA, USA) as Niu et al., [10, 11].

Library construction and RNA-Seq were performed at Beijing BioMarker Technologies (Beijing, China) in accordance with the institute’s protocols and are briefly described here. RNA purity was checked using a NanoPhotometer spectrophotometer (IMPLEN, CA, USA). RNA concentration was measured by the Qubit RNA Assay Kit in a Qubit 2.0 Flurometer (Life Technologies, CA, USA). RNA integrity was assessed using the RNA Nano 6000 Assay Kit of the Agilent Bioanalyzer 2100 system (Agilent Technologies, CA, USA). A total amount of 1 mg RNA per sample was used as input material for the RNA sample preparations. Sequencing libraries were generated using the NEBNext UltraTM RNA Library Prep Kit for Illumina (NEB, USA) following the manufacturer’s recommendations and index codes were added to identify sequences for each sample. mRNA was isolated by NEBNext Poly (A) mRNA Magnetic Isolation Module (NEB, E7490). The cDNA library was constructed following the manufacturer’s instructions of NEBNext Ultra RNA Library Prep Kit for Illumina (NEB, E7530) and NEBNext Multiplex Oligos for Illumina (NEB, E7500). In brief, the enriched mRNA was fragmented into approximately 200 nt RNA inserts, which were used to synthesize the first-strand cDNA and the second cDNA. The double-stranded cDNA was performed end- repair/dA-tail and adaptor ligation. The suitable fragments were isolated by Agencourt AMPure XP beads (Beckman Coulter, Inc.). Then 3 μl USER Enzyme (NEB, USA) was used with size-selected, adaptor-ligated cDNA at 37 °C for 15 min followed by 5 min at 95 °C before PCR. Then PCR was performed with Phusion High-Fidelity DNA polymerase, Universal PCR primers and Index (X) Primer. At last, PCR products were purified (AMPure XP system) and library quality was assessed on the Agilent Bioanalyzer 2100 system. Finally, the cDNA libraries of the chicken (*Gallus gallus*) were sequenced on a flow cell using an Illumina HiSeq™ 2500 sequencing platform.

### Transcriptome analysis using reference genome-based reads mapping

Low quality reads, such as adaptor sequences, unknown nucleotides > 5%, or low Q-value (≤ 20) base more than 20%, were removed by perl script. The clean reads that were filtered from the raw reads were mapped to chicken genome (*Gallus gallus 5.0*) using Tophat2 [59] software. The aligned records from the aligners in BAM/SAM format were further examined to remove potential duplicate molecules. Gene expression levels were estimated using FPKM values (fragments per kilo base of exon per million fragments mapped) by the Cufflinks software [60].

### Identification of differential gene expression

The differential expression analysis of two groups was performed using the DESeq R package (1.10.1). DESeq [61] provide statistical routines for determining differential expression in digital gene expression data using a model based on the negative binomial distribution. The resulting *P* values were adjusted using the Benjamini and Hochberg’s approach for controlling the false discovery rate. Genes with an adjusted *P*-value < 0.05 found by DESeq were assigned as differentially expressed.

### Sequence annotation

Genes were compared against various protein database by BLASTX, including the National Center for Biotechnology Information (NCBI) non-redundant protein (Nr) database, Swiss-Prot database with a cut-off E-value of 10–5. Furthermore, genes were searched against the NCBI non-redundant nucleotide sequence (Nt) database using BLASTn by a cut-off E-value of 10–5. Genes were retrieved based on the best BLAST hit (highest score) along with their protein functional annotation. To annotate the gene with gene ontology (GO) terms, the Nr BLAST results were imported into the Blast2 GO program [62]. GO annotations for the genes were obtained by Blast2 GO. This analysis mapped all the annotated genes to GO terms in the database and counted the number of genes associated with each term. Perl script was then used to plot GO functional classification for the unigenes with a GO term hit to view the distribution of gene functions. The obtained annotation was enriched and refined using TopGo (R package). The gene sequences were also aligned to the Clusters of Orthologous Group (COG) database to predict and classify functions [63]. KEGG pathways were assigned to the assembled sequences by Perl script.

### Confirmation using quantitative real-time PCR

To validate the transcriptome sequencing data, 20 differentially expressed genes related to angiogenesis were selected from potential pathway on 2, 6 and 15 days for Real-time PCR, (*n* = 3, same as RNA-seq) using a TaKaRa SYBR Premix Ex Taq TM II (RR820A; Takara Bio Inc., Dalian, China) by the Quant Studio™ 6 (Applied Biosystems, America). Primer sequences, annealing temperature, and accession numbers are shown in Table 1. Reactions contained 1 μL of cDNA, 0.15 μL of each forward and reverse primer (10 pmol/μL), 6 μL 1 × SYBR Premix Ex Taq II, 0.1 μL ROX dye II and 2.6 μL double distilled H_2_O. A template of each group was amplified by using the following protocol: 95 °C for 3 min, followed by 42 cycles of 95 °C for 30 s, 55 °C for 30 s, and extension at 72 °C for 10 s. 18S rRNA was used as the house-keeping gene, the control samples were used as the calibrator, and the expression of each gene is reported as fold change relative to the control group. The qPCR analysis was performed with three biological and technical replicates. The real-time PCR data were analyzed using the 2^−ΔΔCt^ method. JMP (version 10) program was used for statistical analysis.

**Table 1:** Primers used in real-time PCR analysis

Genes	Primers	Size (BP)	Annealing T (°C)	Accession N0.
*F13*A1	F: GGATGCTTTTGGTCTGATAC	100	55 °C	NM_204685.1
	R: CTGCTTGTTGATCTCATTGG			
*CLU*	F: CGTCAGTTCGGTTGGGTCTT	100	55 °C	NM_204900.1
	R: CCTCGAGGTTGGGAGTTTTG			
*TBXA2R*	F: GACAGCGAAGTGGAGATGAT	100	55 °C	XM_015299775.1
	R: GGAGAGTGGTCTGGATGATG			
*IL1R1*	F: TTTCACGCACCATGAATCTG	100	55 °C	NM_205485.1
	R: GAGCCGAGTTCCACTTCAAT			
*RPL*17	F: GTGAACAAGGCTCCCAAAAT	100	55 °C	NM_001282277.1
	R: CGGTGAGGATCATCTCAATG			
*ITGB3*	F: TTTGTGGACAAGCCCATTTC	100	55 °C	NM_204315.1
	R: CAAACATGGGCAAGCACTTT			
*ITG*AV	F: TCAGTGGTTTGGAGCATCTG	100	55 °C	NM_205439.1
	R: AGGCTCTCGCTCTTGTTTTG			
*ITG*B2	F: CAGTGCGTTCAGCAATAAGA	100	55 °C	NM_205251.1
	R: AGCACGACGAAACTTCACAT			
*RAC2*	F: GGAGAATACATCCCCACTGT	100	55 °C	NM_001201452.1
	R: CCTCTTGTCCAGCAGTATCC			
*ITG*A2	F: TGCTAATAATCCCCGTGTAG	100	55 °C	XM_015277561.1
	R: CAGATCTCCTCCCTGTTGAA			
*IQGAP2*	F: CTGGTGAGGCCAGTAAATTG	100	55 °C	NM_001277778.1
	R: CGCAAACTCTGAATCGATGA			
*GIT1*	F: CATCACGCTGCAGGAGTACCT	100	55 °C	NM_204296.1
	R: CTCATCGCTCAGGCTGTTGT			
*VAV1*	F: CTGGAGACGTTGTAGGGTATG	100	55 °C	NT_469922.1
	R: TGGTGAGACGGGTTGGTT			
*ITGA5*	F: CTCCAACTACCCCGAGTACT	100	55 °C	KC439457.1
	R: CACCGAATAGCCCATATAAC			
*RAP*1B	F: TCGAGTCAAAGACACTGATG	100	55 °C	NM_001007852.1
	R: TAGGTTCTGACCTTGTTCCT			
*FYN*	F: AGGAGTGGTACTTCGGCAAA	100	55 °C	NM_205349.2
	R: GTTTCACTTTCCCGGATCAG			
*PTPN11*	F: TGGAGGCAGAAAATCTACTG	100	55 °C	NM_204968.1
	R: CGCCTAACAGAGAGAGTGAA			
*PTCH1*	F: TTCCTTCTAGCCCATGCGTTT	100	55 °C	NM_204960.2
	R: CTACACTTGCTCCTGTTCGCTTT			
*NCOR2*	F: GGTCCCACACACTTGAC	100	55 °C	XM_015275621.1
	R: GGTGGATACTAGGATGGATT			
*MAML3*	F: GCAACCACACGCTGATCATG	100	55 °C	XM_015276692.1
	R: CCATCGCAAACTCCATTCTG			

### Statistical analysis

Data are presented as mean and standard deviation (Mean ± SD). The value of *P* < 0.05 was considered statistically significant. The quantification data of microscopic figures was obtained with ImageJ 1.42q software and analyzed by on way ANOVA and student *t*-test to compare the differences between mean values of different groups.

## Supplementary information

**Additional file 1.** List of total DEGs on day 2, 6 and 15.

**Additional file 2: Table S1.** Commonly observed differentially expressed genes on day 2, 6 and 15

**Additional file 3: Table S2.** Differentially expressed genes in control and thiram groups on day 2, 6 and 15

**Additional file 4: Figure S1.** Vascularization and calcification. The histopathological observation of tibial growth plate (GP). Calcification and normal blood vessels in the control groups and damaged blood vessels in the treatment groups can clearly be seen in this figure. The relative blood vessels area and erythrocytes were determined with ImageJ software (Bar = 200 μm). **Figure S2.** Quantification of H&E and IHC. **A** The relative area of blood vessels determined in percentage by dividing area of blood vessels with total area (Pixels 4080 × 3072). **B** The density of blood vessels was measured using ImageJ 1.42q software to find denser erythrocytes ratio in control and thiram groups. **C** IHC profile of ImageJ software was used to determine the ITGAV and CLU expression score in control and thiram treated groups. BV = blood vessels; DV = destruction of blood vessels. (Bar-100 μm). 2 DC = 2 days control, 2DT = 2 days treatment, 6 DC = 6 days control, 6DT = 6 days treatment, 15 DC = 15 days control, 15DT = 15 days treatment, C = control, T = treatment, % = percentage. The quantification data of H&E staining and IHC was obtained by ImageJ software. ∗ *P* < 0.05; ∗∗ *P* < 0.01. **Figure S3.** Quantification of IHC. Immunohistochemical localization of ITGAV and CLU in control and thiram treated groups. The results were analyzed by ImageJ software using IHC profile plugin. **Figure S4.** The scatter plot of DEGs enriched in KEGG pathways. The rich factor represents the ratio of DEGs and all unigenes numbers in the pathways; the Q value represents the corrected *P*-value. C = control, T = thiram.

## Data Availability

All data generated or analyzed during this study are included in this published article and its supplementary information files. The datasets are also available from the corresponding author on reasonable request.

## Abbreviations

*ATP2*A3: Sarcoplasmic/endoplasmic reticulum calcium ATPase 3
AvBDs: Avian β-defensins
*CCSAP*: Centriole cilia and spindle-associated protein
CLU: Clusterin precursor
DEGs: Differentially expressed genes
*F13*A1: Coagulation factor XIII A chain protein
*FYN*: Tyrosine-protein kinase Fyn-like
*GIT*1: ARF GTPase-activating protein
GO: Gene ontology
HDPs: Host defense peptides
*IL*1R1: Interleukin-1 receptor type 1 precursor
*IQGAP*2: IQ motif containing GTPase activating protein 2
*ITG*A2: Integrin alpha-2
*ITG*A5: Integrin alpha-IIb-like
*ITG*AV: Integrin alpha-v precursor
*ITG*B2: Integrin beta-2 precursor
*ITG*B3: integrin beta-3 precursor
KEGG: Kyoto Encyclopedia of Genes and Genomes
LEAP-2: Liver-expressed antimicrobial peptide 2
*MAML*3: Mastermind-like protein 3
MAPK: Mitogen-activated protein kinase
MDV: Mareks disease virus
*NCOR*2: Nuclear receptor corepressor 2
NK: Natural killer
*PTCH*1: Protein patched homolog 1
*PTPN*11: Tyrosine-protein phosphatase non-receptor type 11
*RAC*2: Ras-related C3 botulinum toxin substrate 2
*RAP*1B: Ras-related protein Rap-1b precursor
*RASA3*: Ras GTPase-activating protein 3
*RP*L17: Ribosomal protein L17
*SHROOM*2: Shroom2 isoform X6
*TBX*A2R: Thromboxane A2 receptor
TD: Tibial dyschondroplasia
Thiram: Tetramethyl thiuram disulfide
*UBE2*R2: Ubiquitin-conjugating enzyme E2 R2
*VAV*1: Proto-oncogene vav
VEGF: Vascular endothelial growth factor signaling pathway
VEGFR2: Vascular endothelial growth factor receptor 2
