# Supplementary figures and images for: Transcriptome-based screening of intracellular pathways and angiogenesis related genes at different stages of thiram induced tibial lesions in broiler chickens
Source: BMC Genomics. 2020 Jan 15;21:50. doi: 10.1186/s12864-020-6456-9 (PMC6964038; doi:10.1186/s12864-020-6456-9)

**Figure S1**

**
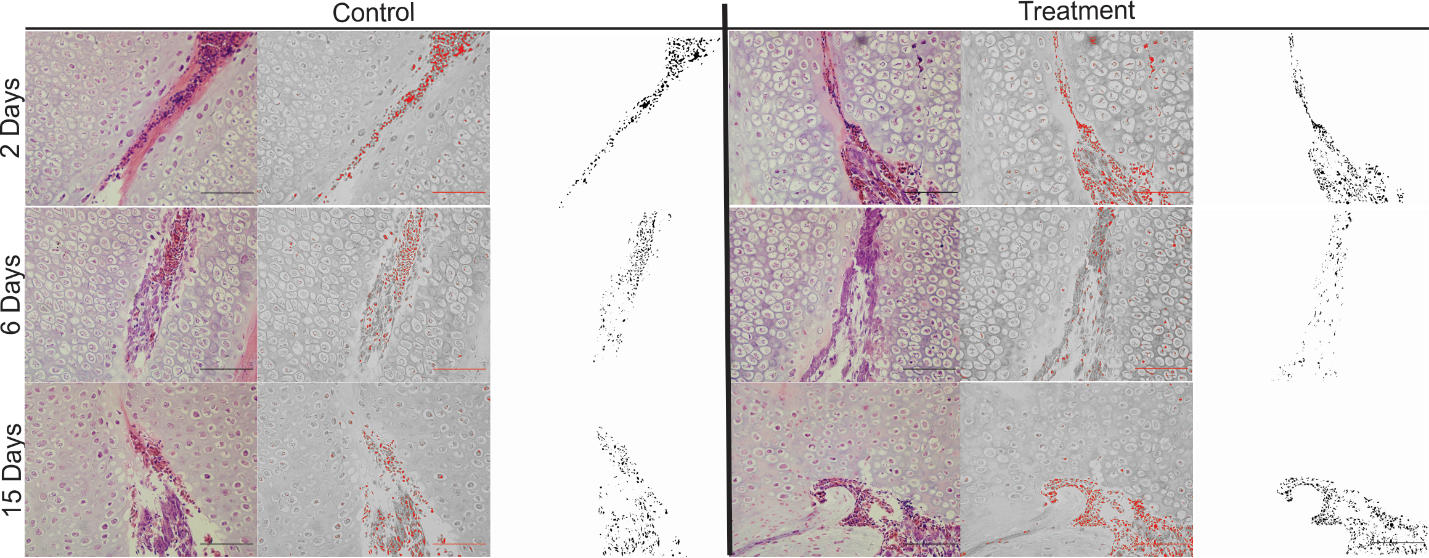
**

**Figure S2**

**
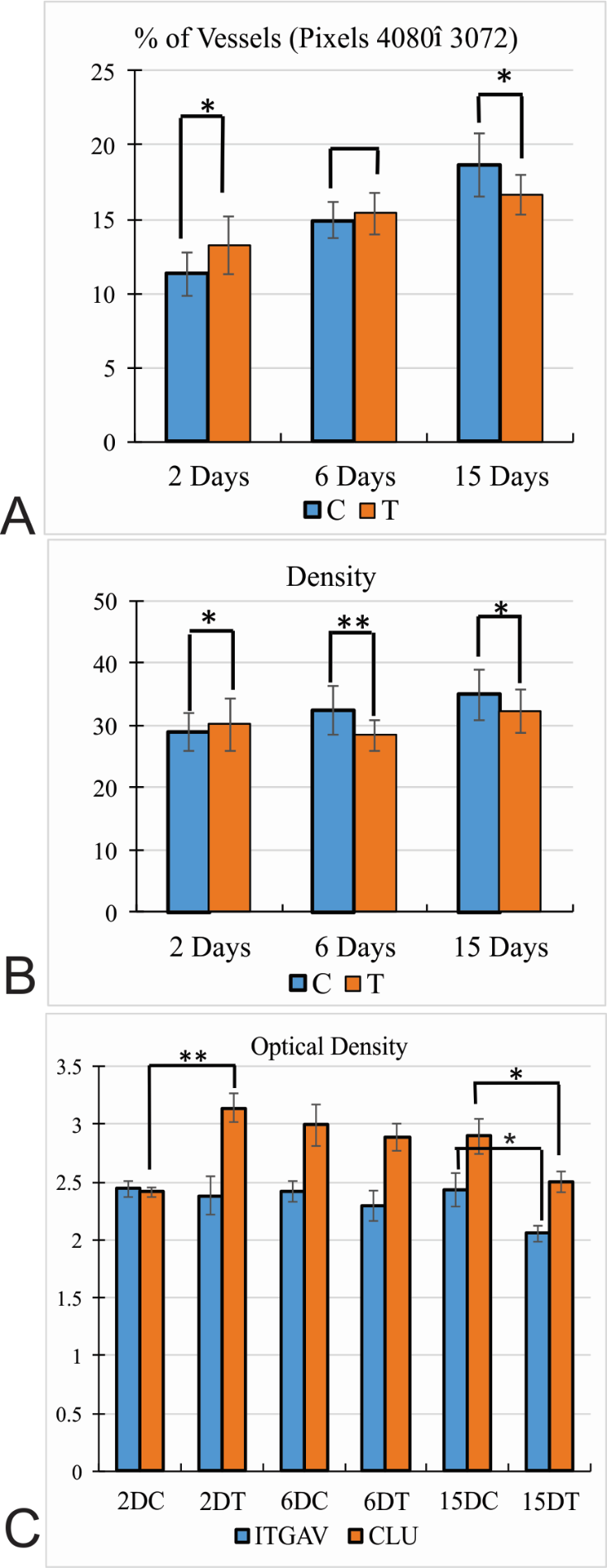
**

**Figure S3**


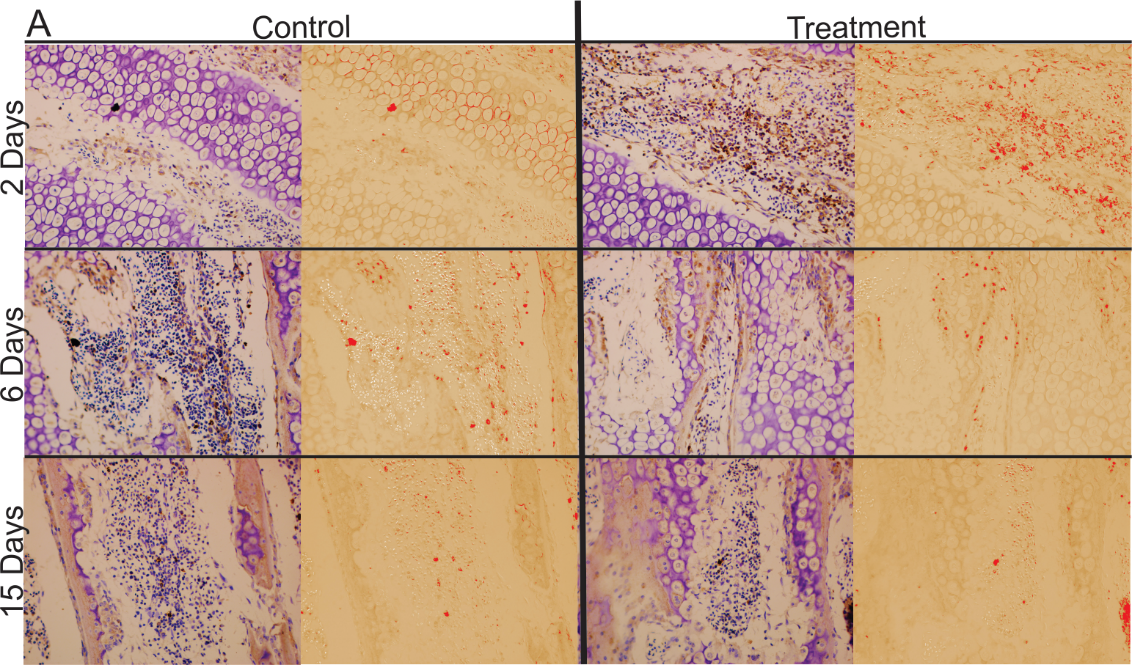


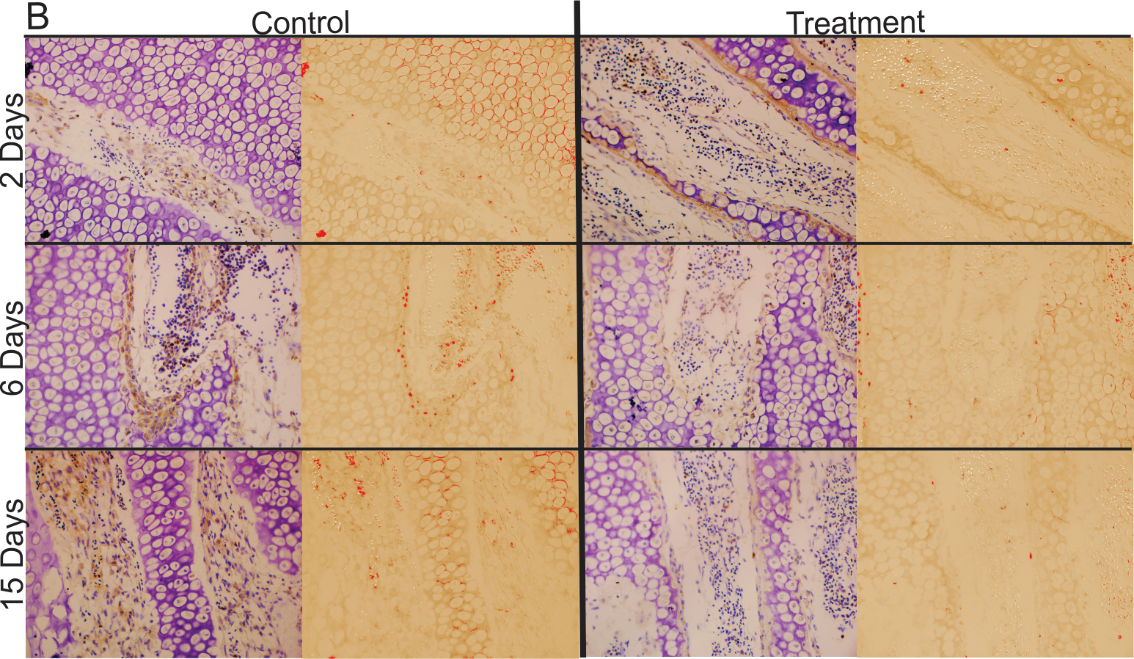


**Figure S4**


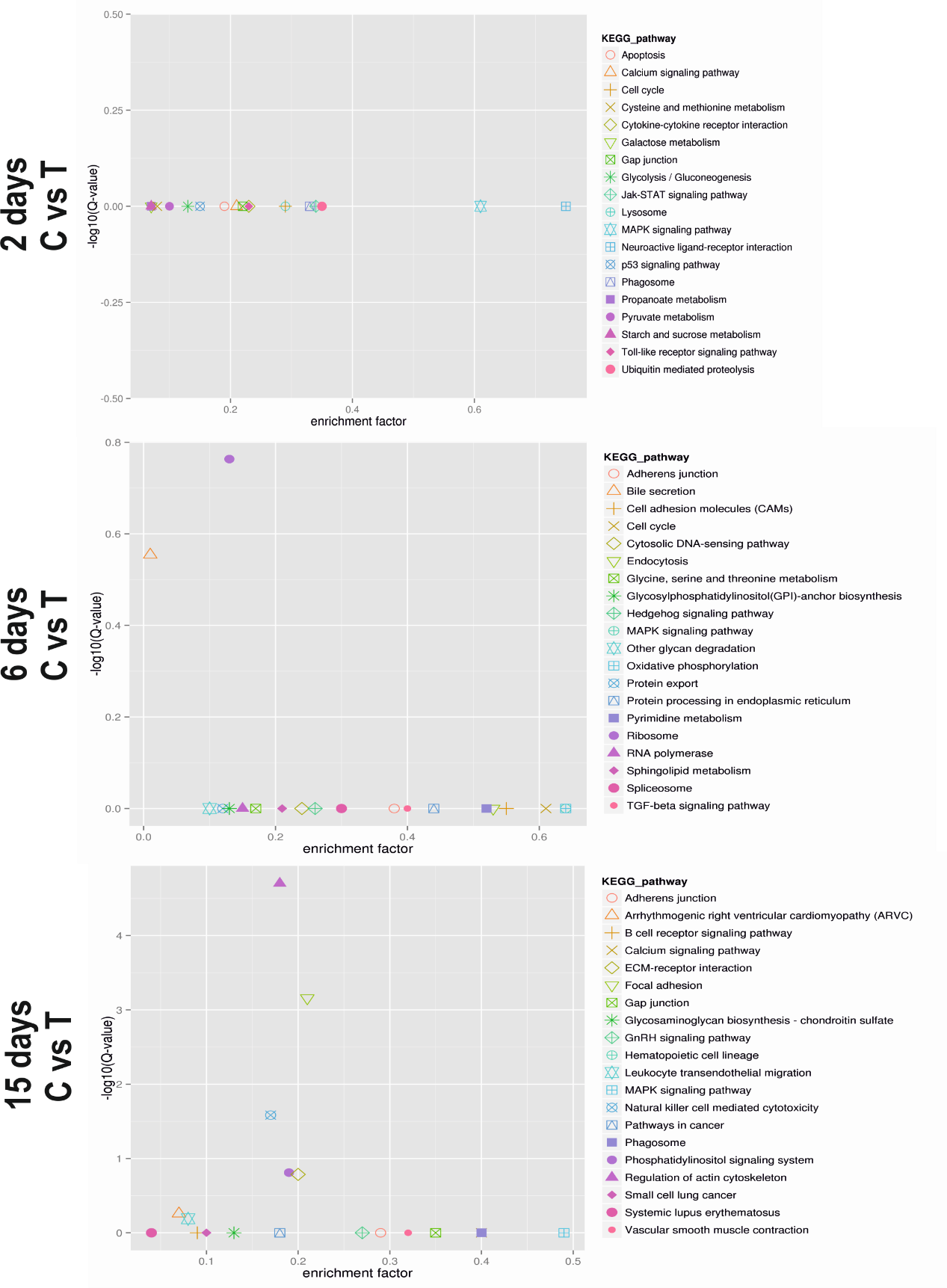

Supplement: Supplementary file 4 — Additional file 4: Figure S1. Vascularization and calcification. The histopathological observation of tibial growth plate (GP). Calcification and normal blood vessels in the control groups and damaged blood vessels in the treatment groups can clearly be seen in this figure. The relative blood vessels area and erythrocytes were determined with ImageJ software (Bar = 200 μm). Figure S2. Quantification of H&E and IHC. A The relative area of blood vessels determined in percentage by dividing area of blood vessels with total area (Pixels 4080 × 3072). B The density of blood vessels was measured using ImageJ 1.42q software to find denser erythrocytes ratio in control and thiram groups. C IHC profile of ImageJ software was used to determine the ITGAV and CLU expression score in control and thiram treated groups. BV = blood vessels; DV = destruction of blood vessels. (Bar-100 μm). 2 DC = 2 days control, 2DT = 2 days treatment, 6 DC = 6 days control, 6DT = 6 days treatment, 15 DC = 15 days control, 15DT = 15 days treatment, C = control, T = treatment, % = percentage. The quantification data of H&E staining and IHC was obtained by ImageJ software. ∗ P < 0.05; ∗∗ P < 0.01. Figure S3. Quantification of IHC. Immunohistochemical localization of ITGAV and CLU in control and thiram treated groups. The results were analyzed by ImageJ software using IHC profile plugin. Figure S4. The scatter plot of DEGs enriched in KEGG pathways. The rich factor represents the ratio of DEGs and all unigenes numbers in the pathways; the Q value represents the corrected P-value. C = control, T = thiram. [file 12864_2020_6456_MOESM4_ESM.docx]
